# The impact of childhood trauma on adolescent depression: the moderating role of sensory processing sensitivity

**DOI:** 10.3389/fpsyg.2025.1678439

**Published:** 2025-11-26

**Authors:** Qianrong Yang, Yachen Tao, Mei Xie, Quanyi Long, Zhi Zhu, Qijun Hu, Meng Zhou, Yuanyuan Zou, Xuzhou Li

**Affiliations:** 1 Faculty of Education, Yunnan Normal University, Kunming, China; 2 Faculty of Nursing, Kunming Medical University, Kunming, China; 3 Faculty of Psychology, Tianjin Normal University, Tianjin, China; 4 Department of Psychology of Developmental and Socialization Processes, Sapienza University of Rome, Rome, Italy; 5 Department of Neurosciences, Mental Health and Sensory Organs—NESMOS, Sapienza University of Rome, Rome, Italy

**Keywords:** childhood trauma, adolescents, depression, sensory processing sensitivity, moderating role

## Abstract

This study investigated the relationship between childhood trauma and adolescent depression, with a particular focus on the moderating role of sensory processing sensitivity (SPS) and its specific subdimensions. A cross-sectional survey was conducted among 843 high school students in China, utilizing the Childhood Trauma Questionnaire (Short Form), the 21-item Highly Sensitive Child Scale, and the Center for Epidemiological Studies Depression Scale. Results indicated that childhood trauma significantly predicted depressive symptoms in adolescents. Moreover, SPS moderated the association between childhood trauma and depression, with higher SPS amplifying the adverse effects of trauma. Further analyses revealed that the three SPS dimensions—Ease of Excitation (EOE), Low Sensory Threshold (LST), and Aesthetic Sensitivity (AES)—each demonstrated unique moderating effects. These findings advance current understanding by illustrating how distinct facets of sensitivity shape vulnerability to depression following childhood trauma. The results offer theoretical contributions to developmental psychopathology and suggest practical implications for designing individualized, sensitivity-informed interventions to support adolescents at risk of depression.

## 1 Introduction

Adolescent depression has become a pressing global public health concern due to its rising prevalence, early onset, and long-term consequences for psychological development and functioning. Adolescence is a critical developmental period characterized by heightened emotional reactivity, identity formation, and increased vulnerability to psychosocial stressors (Rikard-Bell et al., 2022). Depressive symptoms that emerge during this stage often serve as early indicators of future mental health disorders, including major depression, anxiety, and suicidal ideation (Bernaras et al., 2019; Zhou et al., 2020).

Epidemiological research consistently shows that the risk of depression increases markedly in adolescence, with global prevalence rates reaching as high as 34% among individuals aged 10–19 (Shorey et al., 2022). A systematic review by Moreno-Agostino et al. (2021) confirmed an upward trend in both the incidence and burden of depression among youth worldwide. In China, the 2022 National Depression Report estimated that 15–20% of adolescents exhibit significant depressive symptoms, with approximately 41% of affected students reporting school dropout as a consequence of poor mental health (Cui et al., 2024).

The consequences of adolescent depression are multifaceted. On an individual level, depression impairs cognitive functioning, academic performance, and social integration, while increasing the risk for substance abuse and self-harm (Cairns et al., 2014; Copeland et al., 2018). On a broader scale, untreated adolescent depression contributes to a substantial burden on educational and healthcare systems, making early identification and prevention efforts particularly urgent.

Despite the growing attention to adolescent depression, its etiology remains complex and multifactorial, involving interactions between early life stressors, such as childhood trauma, and individual vulnerability traits (Engel-Yeger et al., 2016). Accordingly, contemporary developmental psychopathology emphasizes the importance of examining both environmental risk factors and person-specific moderators to better understand divergent developmental outcomes (Jeon and Bae, 2022; Mandelli et al., 2015; Uher, 2014). Investigating how adolescents’ sensitivity to environmental stimuli might shape their psychological responses to early adversity is thus critical for advancing both theory and clinical practice. Nevertheless, despite extensive research linking childhood trauma to adolescent depression, several conceptual and empirical gaps remain. Prior studies have primarily examined the direct effects of childhood adversity on depression, often overlooking the moderating role of individual sensitivity traits that may explain why some adolescents develop depressive symptoms while others do not. Moreover, although sensory processing sensitivity (SPS) has been identified as a potential susceptibility factor, most existing work has focused on general life stressors or parenting quality rather than comprehensive, multidimensional trauma experiences. In addition, past research has typically treated SPS as a unidimensional construct, neglecting the distinct influences of its subcomponents—Ease of Excitation, Low Sensory Threshold, and Aesthetic Sensitivity—on mental health outcomes. Empirical evidence on these mechanisms also remains scarce in non-Western adolescent populations, limiting the cultural generalizability of current models. The present study addresses these gaps by examining SPS as a moderator in the relationship between multidimensional childhood trauma and adolescent depression, adopting a multidimensional approach to SPS to reveal differential vulnerability patterns, and extending this line of research to a large Chinese adolescent sample. By integrating environmental and dispositional factors within a developmental psychopathology framework, this study advances theoretical understanding and provides culturally grounded implications for individualized, sensitivity-informed mental health interventions.

### 1.1 Childhood trauma and adolescent depression

Childhood trauma refers to experiences of abuse, neglect, or other adverse events that occur during early developmental stages and are typically perpetrated by caregivers or other trusted individuals. These events can result in lasting harm to a child’s physical safety, emotional security, or psychosocial development (Cassell, 2013; Karaca Dinç et al., 2021; Zhang et al., 2018). Common forms of childhood trauma include physical, emotional, and sexual abuse, as well as physical and emotional neglect (Qin et al., 2024). Numerous studies have demonstrated that childhood trauma has a detrimental impact on adolescent development, particularly by undermining self-worth, cognitive functioning, and affect regulation capacities (Gerin et al., 2024; Kerley et al., 2023; Knipschild et al., 2024).

Theoretical models such as the diathesis–stress framework suggest that early trauma may sensitize individuals to later stressors, increasing their susceptibility to internalizing disorders such as depression (Benham, 2024). Empirical research consistently supports this association: adolescents with a history of childhood trauma are at elevated risk for depressive symptoms, anxiety, suicidality, and behavioral problems compared to their non-traumatized peers (Copeland et al., 2018; Wei and Lü, 2023). A large-scale burden-of-disease analysis in China further identified childhood abuse and sexual violence as leading contributors to adolescent depression (Zhou et al., 2020), reinforcing the significance of childhood trauma as a public mental health concern (Burgard et al., 2022). However, not all individuals exposed to early trauma develop depression, highlighting the need to consider moderating factors that account for such variability in outcomes.

### 1.2 The moderating role of sensory processing sensitivity

Interindividual variability in the psychological impact of childhood trauma suggests that certain personality traits may modulate susceptibility to depression. One such trait is Sensory Processing Sensitivity (SPS), a genetically influenced temperamental characteristic defined by heightened responsiveness to both external and internal stimuli (Aron et al., 2012; Greven et al., 2019; Pluess and Boniwell, 2015). Individuals high in SPS tend to process sensory and emotional information more deeply, show greater emotional reactivity, and are more easily overwhelmed by intense environments (Aghaeimazraji et al., 2024; Acevedo et al., 2018; Liss et al., 2005).

From a differential susceptibility perspective, SPS has been conceptualized as a “plasticity” factor—amplifying both the negative impact of adverse experiences and the positive benefits of supportive contexts (Pluess, 2015). Adolescents with high SPS have been shown to derive more benefit from therapeutic interventions and nurturing relationships, but they also report increased distress and psychopathology in the presence of environmental adversity (Aron et al., 2012; Greven et al., 2019). In particular, SPS has been linked to a greater risk of depression under conditions of low parental warmth or high family conflict (Cox et al., 2024; Gross et al., 2017; Liss et al., 2005). However, under high-quality caregiving, high-SPS adolescents often demonstrate resilience and well-being equivalent to or exceeding that of their low-SPS counterparts.

Although previous research has explored how parenting or general life stress interacts with SPS to influence adolescent mental health, relatively little is known about whether SPS moderates the specific relationship between childhood trauma and depression. While Wei and Lü (2024) examined the moderating role of SPS in the link between childhood abuse and depressive symptoms, their study focused on a single dimension of abuse rather than a multidimensional trauma framework. The present research extends this line of inquiry by examining broader trauma experiences and multiple SPS subdimensions, thereby contributing a novel and more comprehensive understanding of this relationship. Given that SPS encompasses multiple dimensions—including Ease of Excitation (EOE), Low Sensory Threshold (LST), and Aesthetic Sensitivity (AES)—a multidimensional approach may reveal more nuanced patterns of vulnerability or resilience in the face of early trauma. Investigating the moderating role of SPS in the childhood trauma–depression relationship is thus essential to understanding individual variability in psychological outcomes and may inform personalized intervention strategies.

### 1.3 Research objectives and hypotheses

In light of the evidence reviewed, several conceptual and empirical gaps remain unaddressed. Previous research has primarily focused on the direct effects of childhood trauma on adolescent depression, overlooking the moderating influence of individual sensitivity traits that could explain interindividual variability in depressive outcomes. Moreover, although sensory processing sensitivity (SPS) has been proposed as a potential susceptibility factor, few studies have examined its role within the specific context of *multidimensional childhood trauma*, and even fewer have investigated the distinct moderating effects of its subcomponents—Ease of Excitation (EOE), Low Sensory Threshold (LST), and Aesthetic Sensitivity (AES). In addition, existing findings are largely derived from Western samples, leaving a paucity of cross-cultural evidence on how SPS functions in non-Western adolescent populations.

To address these gaps, the present study aims to advance current knowledge by (a) examining whether SPS moderates the association between childhood trauma and adolescent depression, (b) testing whether different SPS dimensions exert differential moderating effects, and (c) extending this research to a large sample of Chinese adolescents to enhance the cross-cultural validity of SPS theory. By focusing on adolescents—a population particularly vulnerable to both trauma and depressive outcomes—this research contributes to developmental psychopathology literature and offers practical implications for early screening and sensitivity-informed interventions.

**Figure 1:**
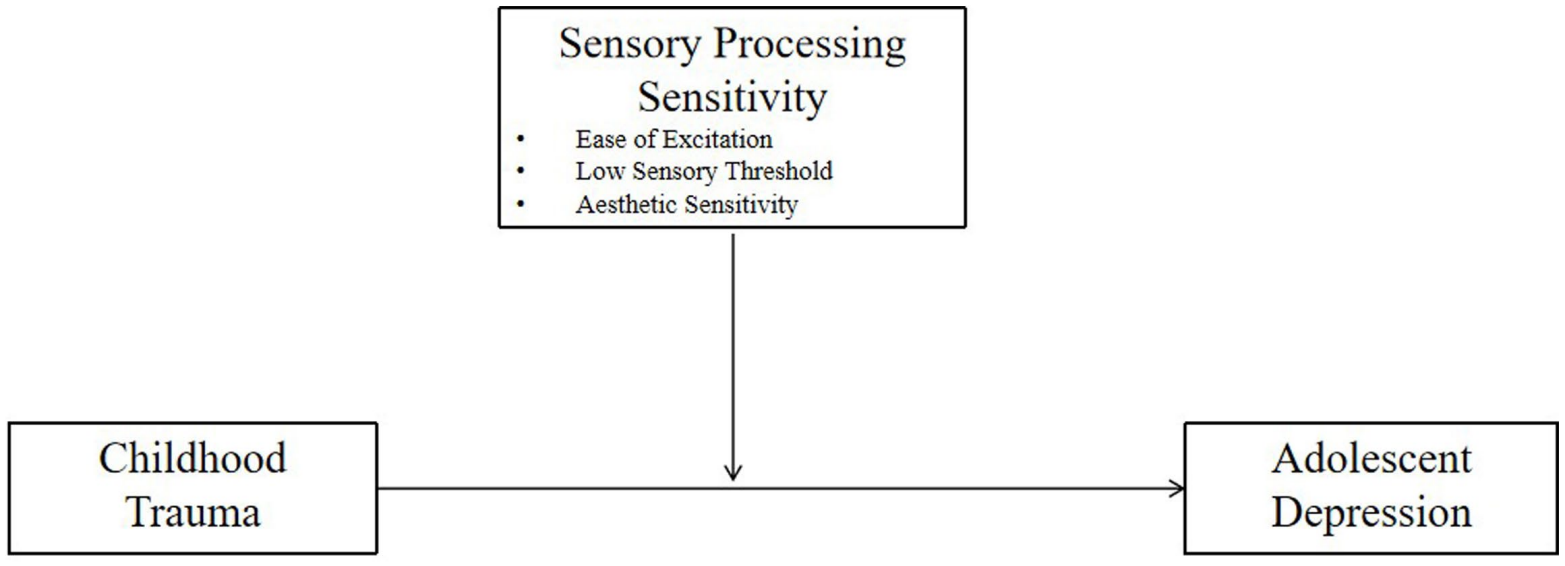
The proposed theoretical model.

The proposed theoretical model is shown in Figure 1. The study tests the following hypotheses:

*H*1: Childhood trauma is positively associated with adolescent depression. Adolescents with higher levels of childhood trauma exposure are more likely to report elevated depressive symptoms.

*H*2: Sensory processing sensitivity moderates the relationship between childhood trauma and adolescent depression. Adolescents with higher SPS levels will show a stronger positive association between childhood trauma and depressive symptoms than those with lower SPS.

*H*3: The subdimensions of SPS (EOE, LST, AES) differentially moderate the childhood trauma–depression relationship, suggesting that specific facets of sensitivity play distinct roles in shaping adolescents’ vulnerability or resilience to early trauma.

## 2 Methods

### 2.1 Participants

This study employed a stratified cluster sampling method to recruit participants. Students were randomly selected from three classes in each grade level (Grades 10 to 12) across two high schools in Yunnan Province, yielding a total of 18 participating classes. Based on established psychometric guidelines recommending a sample size of 5 to 10 participants per questionnaire item (Costello and Osborne, 2005), and given the total of 69 items included in the survey instruments, the estimated minimum required sample size ranged from 345 to 690. To account for potential issues such as non-responses, data quality, and sampling error, a total of 846 questionnaires were distributed to ensure adequate statistical power and representativeness.

The survey was conducted in classroom settings, with standardized instructions read aloud by trained research assistants to ensure consistency across sessions. Each session lasted approximately 15 min. Prior to data collection, written informed consent was obtained from all participants, and participation was entirely voluntary. Of the 846 questionnaires distributed, 843 were returned (response rate = 99.6%). After excluding 9 questionnaires due to excessive missing data, 834 valid responses were retained for analysis, yielding an effective response rate of 98.6%.

Demographic information was collected across 15 variables. Among the participants, 292 were in Grade 10 (35%), 284 in Grade 11 (34.1%), and 258 in Grade 12 (30.9%). A chi-square test indicated no significant differences across grade levels (*p* > 0.05). The mean age of participants was 16.41 years (*SD* = 0.99). A total of 441 students were from urban areas and 393 from rural areas, with no significant difference between the two groups (*p* > 0.05). However, a significant gender imbalance was observed: 340 participants were male and 494 were female (*p* < 0.05). As a result, gender was included as a control variable in subsequent regression analyses. All research procedures were reviewed and approved by the Institutional Review Board (IRB) of the first author’s affiliated university.

### 2.2 Measures

To ensure the reliability and validity of the research instruments, this study employed widely recognized and psychometrically robust scales adapted from leading international studies. The survey consisted of three sections assessing the core constructs of interest: childhood trauma, sensory processing sensitivity (SPS), and depressive symptoms.

#### 2.2.1 Childhood trauma

Childhood trauma was measured using the Childhood Trauma Questionnaire–Short Form (CTQ-SF), a self-report instrument developed by Bernstein et al. (1998) to assess adverse experiences before the age of 16. The Chinese version, revised by Zhao et al. (2005) and validated by Zhang (2011), has demonstrated strong cultural applicability and psychometric properties. The CTQ-SF comprises 28 items across five dimensions: emotional abuse, physical abuse, sexual abuse, emotional neglect, and physical neglect. Responses are rated on a 5-point Likert scale ranging from 1 (“never true”) to 5 (“very often true”), with higher scores indicating more severe exposure to childhood trauma. In the current study, the Cronbach’s alpha coefficient for the total scale was 0.682, indicating excellent internal consistency.

#### 2.2.2 Sensory processing sensitivity

SPS was assessed using the 21-item Highly Sensitive Child Scale (HSC-21), a self-report questionnaire designed to capture individual differences in environmental sensitivity among children and adolescents. The original scale was developed by Pluess et al. (2018) and later refined by Weyn et al. (2022). This study employed the Chinese version adapted by Jiang and Tao (2023), which has shown good reliability and validity in Chinese middle school populations. The scale contains three dimensions: Ease of Excitation (EOE), Low Sensory Threshold (LST), and Aesthetic Sensitivity (AES). Items are rated on a 7-point Likert scale ranging from 1 (“strongly disagree”) to 7 (“strongly agree”), with higher scores reflecting greater sensitivity to environmental stimuli. The Cronbach’s alpha for the overall SPS scale was 0.841, with subscale alphas of 0.737, 0.755, and 0.771, demonstrating satisfactory reliability.

#### 2.2.3 Depression

Depressive symptoms were assessed using the Center for Epidemiologic Studies Depression Scale (CES-D), originally developed by Radloff (1977) to measure the frequency of depressive symptoms in community populations. The Chinese version, revised by Chen et al. (2009), has been validated among adolescents and demonstrates high internal consistency and construct validity. It also exhibits satisfactory criterion validity and diagnostic sensitivity, effectively distinguishing between clinical and non-clinical levels of depressive symptoms at the cutoff score of 16 (Chen et al., 2009).

The CES-D comprises 20 items rated on a 4-point Likert scale ranging from 0 (“rarely or none of the time”) to 3 (“most or all of the time”). It covers four domains: depressed affect, positive affect (reverse scored), somatic complaints, and interpersonal problems. Total scores range from 0 to 60, with higher scores indicating more severe depressive symptoms; a score of 16 or above suggests clinically significant depression. In the present study, the CES-D demonstrated excellent reliability, with a Cronbach’s alpha of 0.883, consistent with previous findings among Chinese adolescent samples.

### 2.3 Data analysis

Data were first entered using EpiData 3.1 and subsequently cleaned and analyzed using SPSS 26.0. Descriptive statistics were computed to summarize the demographic characteristics and distribution of key variables. Pearson correlation analyses were conducted to examine bivariate relationships among childhood trauma, sensory processing sensitivity (SPS), and depressive symptoms. To test the study’s hypotheses, particularly the moderating role of SPS, moderated multiple linear regression analyses were performed using Model 1 of the PROCESS macro for SPSS (Hayes, 2017). To probe significant interaction effects, simple slope analysis was conducted. Prior to this analysis, the depression scores were standardized into z-scores to facilitate the interpretation of the interaction. The analysis involved visualizing the relationship between childhood trauma and depression at high (+1 SD) and low (−1 SD) levels of the moderator (SPS and its sub-dimensions; Preacher et al., 2006). Gender was included as a covariate in all regression models due to its significant association with depressive symptoms in the preliminary analyses.

Regarding the analytical strategy for testing the moderating effects, our primary focus was on the total score of Sensory Processing Sensitivity (SPS). The follow-up analyses involving the three sub-dimensions of SPS (EOE, LST, AES) were conducted to provide a nuanced interpretation of the overall effect observed at the total score level.

## 3 Results

### 3.1 Common method bias analysis

Given that all data were collected through self-report questionnaires, Harman’s single-factor test was conducted to assess potential common method variance (CMV; Podsakoff et al., 2003). An unrotated exploratory factor analysis was performed, extracting all factors with eigenvalues greater than 1. Results showed that the first principal component accounted for only 17.657% of the total variance (see Table 1), well below the conventional threshold of 40%, suggesting that common method bias is unlikely to pose a serious threat to the validity of the findings (Hou and Long, 2004).

**Table 1:** Total variance explained (Harman’s single-factor test).

Component	Eigenvalue	% Variance explained	Cumulative %
1	12.183	17.657	17.657
2	5.603	8.121	25.778
3	3.871	5.610	31.388
4	2.517	3.648	35.036
5	2.123	3.077	38.113
6	1.752	2.540	40.653
7	1.661	2.407	43.060
8	1.632	2.365	45.425
9	1.479	2.144	47.569
10	1.389	2.013	49.582
11	1.318	1.911	51.492
12	1.259	1.825	53.317
13	1.209	1.752	55.069
14	1.096	1.589	56.658
15	1.065	1.544	58.202
16	1.061	1.538	59.740

### 3.2 Correlation analysis of variables

As presented in Table 2, significant correlations were observed between depression scores and all three dimensions of sensory processing sensitivity (EOE, LST, AES), the total SPS score, as well as each subscale and the total score of childhood trauma. Specifically, the “positive affect” subscale of the CES-D was negatively correlated with the EOE and LST dimensions and the overall SPS score. It was also negatively associated with all CTQ-SF subscales and total childhood trauma score. All other pairs of variables showed positive correlations, with coefficients greater than zero, indicating that higher scores on childhood trauma and SPS are generally associated with greater depressive symptomatology. These correlation patterns provide preliminary support for the study’s three hypotheses.

**Table 2:** Correlation analysis of variables.

	Grade	Age	Gender	Income	Region	Religion	EOE	AES	LST	SPS	DA	SC	PA	IP	Depression	PN	EA	EN	PAB	SA	CT
Grade	--																				
Age	0.808**	--																			
Gender	0.073*	0.02	--																		
Income	−0.007	0	0.038	--																	
Region	0.096**	0.189**	0.123**	0.152**	--																
Religion	−0.03	−0.007	0.033	0.029	0.048	--															
EOE	−0.059	−0.046	0.135**	0.05	0.055	−0.031	--														
AES	−0.013	−0.025	0.004	−0.124**	−0.101**	−0.039	0.239**	--													
LST	−0.034	−0.003	0.034	0.01	0.012	−0.045	0.572**	0.415**	--												
SPS	−0.046	−0.034	0.077*	−0.031	−0.017	−0.049	0.783**	0.724**	0.825**	--											
DA	−0.066	−0.037	0.150**	−0.006	0.059	−0.019	0.483**	0.170**	0.338**	0.426**	--										
SC	−0.076*	−0.044	0.087*	0.024	0.105**	0.013	0.392**	0.124**	0.287**	0.345**	0.629**	--									
PA	−0.135**	−0.122**	0.070*	0.090**	0.071*	0.043	0.329**	−0.106**	0.133**	0.152**	0.416**	0.326**	--								
IP	−0.094**	−0.046	0.053	−0.022	0.082*	0.043	0.402**	0.079*	0.269**	0.322**	0.723**	0.556**	0.305**	--							
Depression	−0.106**	−0.072*	0.131**	0.022	0.094**	0.013	0.512**	0.113**	0.339**	0.414**	0.906**	0.832**	0.609**	0.762**	--						
PN	−0.037	0.009	0.044	0.107**	0.248**	0.028	0.097**	0.009	0.062	0.072*	0.143**	0.157**	0.049	0.138**	0.154**	--					
EA	−0.043	−0.021	0.128**	0.026	0.120**	−0.024	0.232**	0.063	0.142**	0.189**	0.452**	0.367**	0.230**	0.392**	0.458**	0.311**	--				
EN	−0.143**	−0.075*	−0.052	0.047	0.095**	0.078*	0.126**	−0.070*	0.013	0.031	0.225**	0.178**	0.337**	0.248**	0.290**	0.145**	0.250**	--			
PAB	0.011	0.042	−0.045	0.011	0.101**	−0.013	0.075*	0.025	0.065	0.071*	0.187**	0.144**	0.02	0.172**	0.167**	0.336**	0.473**	0.114**	--		
SA	−0.02	−0.008	−0.001	0.036	0.075*	−0.011	0.082*	0	0.065	0.062	0.105**	0.098**	−0.009	0.069*	0.095**	0.289**	0.387**	−0.02	0.441**	--	
CT	−0.086*	−0.025	0.037	0.071*	0.192**	0.024	0.207**	0.003	0.107**	0.137**	0.375**	0.315**	0.238**	0.347**	0.399**	0.615**	0.771**	0.562**	0.651**	0.556**	--

Income means total monthly income of parents; Region (1 = town, 2 = rural area); Religion (1 = yes, 2 = no); EOE means ease of excitation; LST means low sensory threshold; AES means aesthetic sensitivity, SPS means sensory processing sensitivity; EA means emotional abuse; PAB means physical abuse; SA means sexual abuse; EN means emotional neglect; PN means physical neglect; DA means depressed affect; PA means positive affect; SC means somatic complaints, IP means interpersonal problems, and CT means childhood trauma. *p < 0.05, **p < 0.01.

### 3.3 Moderation analysis of sensory processing sensitivity

#### 3.3.1 Moderating role of overall sensory processing sensitivity

To examine the moderating effect of sensory processing sensitivity (SPS) on the relationship between childhood trauma and adolescent depression, a multiple regression analysis was conducted with childhood trauma as the independent variable, depression as the dependent variable, and SPS as the moderator. Gender was entered as a control variable. As shown in Table 3, the model explained 30.5% of the variance in depression (R^2^ = 0.305). The overall model was statistically significant (*p* < 0.05), indicating good model fit.

**Table 3:** Summary of moderation model coefficients for the effects of childhood trauma, gender, and sensory processing sensitivity (SPS and subdimensions) on depression.

Model	Constant	Gender	Childhood trauma	Moderator	Interaction	R^2^	F	*p*
SPS (total)	−0.301	0.176**	0.262***	0.383***	0.160***	0.305	79.791	0.000
EOE (Ease of Excitation)	−0.238	0.128*	0.240***	0.370***	0.161***	0.280	70.698	0.000
LST (Low Sensory Threshold)	−0.378	0.226**	0.275***	0.324***	0.149***	0.255	62.165	0.000
AES (Aesthetic Sensitivity)	−0.374	0.234**	0.344***	0.214***	0.098**	0.198	44.679	0.000

**p < 0.01, ***p < 0.001. Gender (1 = male, 2 = female).

**Figure 2:**
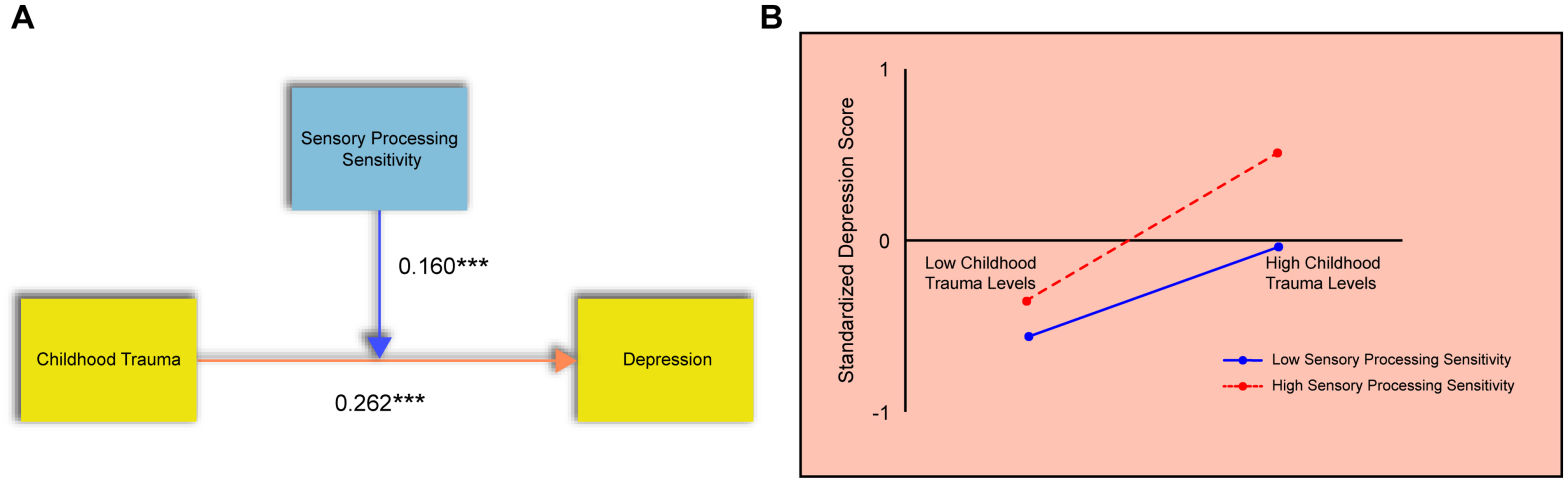
The moderating effect of sensory processing sensitivity. **(A)** Results of the regression analysis showing the significant interaction between childhood trauma and SPS on depression. **(B)** Simple slope analysis. The Y-axis represents standardized depression scores. The lines depict the relationship between childhood trauma (X-axis) and depression at high (+1 SD) and low (−1 SD) levels of SPS.

Regression results showed that both childhood trauma and SPS were significantly and positively associated with depression (*p* < 0.05), with corresponding positive regression coefficients. Moreover, the interaction term (childhood trauma × SPS) was also significant (*p* < 0.05) and positively associated with depression, suggesting that SPS significantly moderates the relationship between childhood trauma and depressive symptoms (Figure 2A). Probing this interaction, the simple slope analysis (Figure 2B) showed that the positive association between childhood trauma and depression was significantly stronger for adolescents with high levels of SPS (+1 SD) than for those with low levels of SPS (−1 SD).

#### 3.3.2 Moderating effect of the “ease of excitation” dimension

A separate regression model was run to examine the moderating role of the EOE subscale of SPS. Childhood trauma was the independent variable, depression the dependent variable, EOE the moderator, and gender was again controlled. The model accounted for 28% of the variance in depression (R^2^ = 0.280), and the regression was statistically significant (*p* < 0.05), as shown in Table 3.

**Figure 3:**
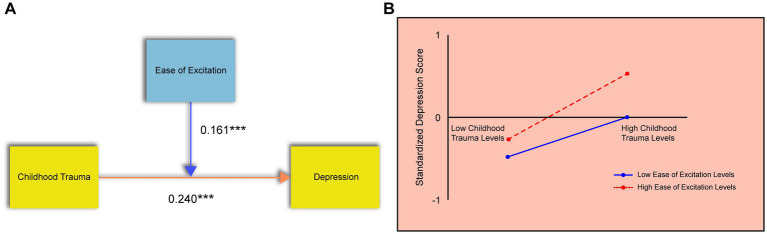
The moderating effect of the ease of excitation dimension. **(A)** Results of the regression analysis showing the significant interaction between childhood trauma and EOE on depression. **(B)** Simple slope analysis. The Y-axis represents standardized depression scores. The lines depict the relationship between childhood trauma (X-axis) and depression at high (+1 SD) and low (−1 SD) levels of EOE.

Both childhood trauma and EOE significantly predicted depression (*p* < 0.05), and the interaction term (childhood trauma × EOE) was also significant with a positive regression coefficient, indicating that EOE strengthens the link between childhood trauma and depression (Figure 3A). Simple slope analysis (Figure 3B) revealed that the positive association between childhood trauma and depression was stronger among adolescents with high levels of EOE. The moderating effect of EOE suggests that individuals with greater emotional reactivity are more sensitive to the negative emotional consequences of early trauma.

#### 3.3.3 Moderating effect of the “low sensory threshold” dimension

To examine whether the Low Sensory Threshold (LST) dimension of sensory processing sensitivity moderates the relationship between childhood trauma and depression, a multiple regression analysis was conducted. Childhood trauma was entered as the independent variable, depression as the dependent variable, LST as the moderator, and gender was included as a control variable.

As shown in Table 3, the regression model explained 25.5% of the variance in depression scores (R^2^ = 0.255), and the overall model fit was statistically significant (*p* < 0.05), indicating that the model was appropriate. The regression coefficients for both childhood trauma and LST were significant and positive (*p* < 0.05), suggesting that each independently contributes to increased depressive symptoms.

**Figure 4:**
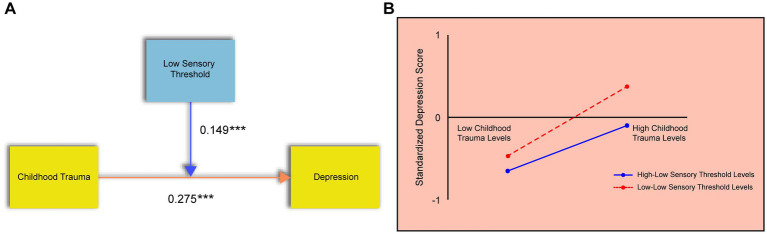
The moderating effect of the low sensory threshold dimension. **(A)** Results of the regression analysis showing the significant interaction between childhood trauma and LST on depression. **(B)** Simple slope analysis. The Y-axis represents standardized depression scores. The lines depict the relationship between childhood trauma (X-axis) and depression at high (+1 SD) and low (−1 SD) levels of LST.

Importantly, the interaction term (childhood trauma × LST) was also statistically significant with a positive coefficient, indicating that LST plays a significant moderating role in the relationship between childhood trauma and depression (Figure 4A). The pattern from the simple slope analysis (Figure 4B) confirmed that adolescents with a high LST were more susceptible to the depressogenic effects of childhood trauma.

#### 3.3.4 Moderating effect of the “aesthetic sensitivity” dimension

A similar moderation analysis was conducted for the Aesthetic Sensitivity (AES) dimension of SPS. Childhood trauma was specified as the independent variable, depression as the dependent variable, AES as the moderator, and gender as the control variable.

The results of the regression model (Table 3) revealed that AES significantly moderates the association between childhood trauma and depression. The model accounted for 19.8% of the variance in depression scores (R^2^ = 0.198), and the overall model was statistically significant (*p* < 0.05). Both childhood trauma and AES showed significant positive regression coefficients (*p* < 0.05), indicating their individual contributions to depression.

**Figure 5:**
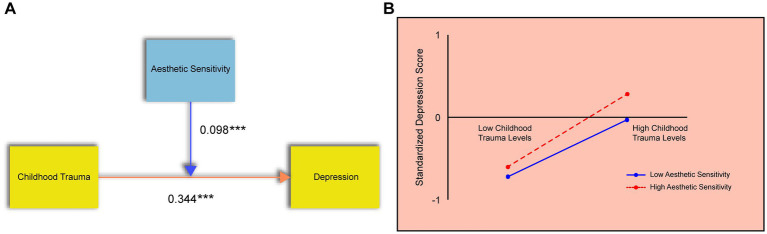
The moderating effect of the aesthetic sensitivity dimension. **(A)** Results of the regression analysis showing the significant interaction between childhood trauma and AES on depression. **(B)** Simple slope analysis. The Y-axis represents standardized depression scores. The lines depict the relationship between childhood trauma (X-axis) and depression at high (+1 SD) and low (−1 SD) levels of AES.

The interaction term (childhood trauma × AES) was also significant and positive, confirming that AES strengthens the relationship between childhood trauma and depressive symptoms (Figure 5A). As illustrated by the simple slopes (Figure 5B), higher aesthetic sensitivity intensified the relationship between childhood trauma and depression.

## 4 Discussion

This study constructed a moderation model to investigate the relationships among childhood trauma, sensory processing sensitivity (SPS), and adolescent depression. The aim was to systematically explore the mechanisms and influencing factors underlying depressive symptoms in adolescents, addressing several gaps in the existing literature. Previous studies have largely focused on the direct association between childhood trauma and depression, with limited attention to how individual sensitivity traits may moderate this relationship. Moreover, prior research has often treated SPS as a single, global construct, neglecting the potentially distinct roles of its subdimensions—Ease of Excitation (EOE), Low Sensory Threshold (LST), and Aesthetic Sensitivity (AES)—in shaping adolescents’ emotional responses to early adversity. In addition, most empirical evidence has been derived from Western contexts, leaving a lack of cross-cultural understanding of how SPS operates in non-Western adolescent populations.

The results of this study help bridge these gaps by demonstrating that childhood trauma is significantly associated with adolescent depression, and that SPS, along with its specific subdimensions, moderates this association in meaningful and differentiated ways. All three hypotheses were supported, providing novel empirical evidence for understanding how childhood trauma contributes to adolescent depression and how individual differences in sensitivity traits influence this process. These findings extend current developmental psychopathology frameworks and offer valuable insights for designing culturally sensitive, individualized intervention strategies to support adolescents at risk of depression.

### 4.1 Association between childhood trauma and adolescent depression

Consistent with Hypothesis 1, this study found that childhood trauma is positively associated with adolescent depression, suggesting that higher levels of traumatic childhood experiences correspond to more severe depressive symptoms (Serafini et al., 2016). This result aligns with previous research showing that early life adversity is strongly linked to damage in the central nervous system and alterations in neurobiological systems. These alterations may include the sensitization of neural circuits and persistent dysregulation of neurotransmitter systems, thereby increasing individuals’ vulnerability to depression and heightening their stress reactivity (Acevedo et al., 2017).

More specifically, childhood trauma has been shown to disrupt the functional activity of the hypothalamic–pituitary–adrenal (HPA) axis, as evidenced by abnormal results in dexamethasone/corticotropin-releasing factor tests and hyperactivity of the HPA axis. This overactivation is considered a potential biological marker of depression (Heim et al., 2004; Heim et al., 2008; Lu et al., 2016). Neuroimaging studies have also revealed that childhood trauma is associated with both structural and functional brain abnormalities in individuals with depression. These include reductions in gray matter volume in areas such as the left dorsolateral prefrontal cortex and abnormalities in other regions critical to mood regulation and stress response (Ahn et al., 2016; Lu et al., 2016; Yang et al., 2017).

In addition to increasing the risk of developing depression, childhood trauma can also alter the clinical presentation of depressive symptoms through its impact on neural architecture and function. Taken together, these findings reaffirm the significant role of childhood trauma in shaping adolescent depressive outcomes and highlight the need to consider its neurobiological underpinnings when developing prevention and intervention strategies.

### 4.2 The moderating role of sensory processing sensitivity

In line with Hypothesis 2, the results revealed that SPS moderates the relationship between childhood trauma and adolescent depression, confirming the relevance of individual sensitivity traits in shaping psychological outcomes. This finding is consistent with the theory of gene–environment interactions, which posits that specific genetic or temperament-based predispositions can magnify individuals’ sensitivity to environmental adversity (Uher, 2014). When such traits coexist with early traumatic experiences, they can jointly elevate the likelihood of developing depression (Mandelli et al., 2015).

SPS, characterized by deep cognitive processing and emotional responsiveness, was found to be positively associated with depression (Wei and Lü, 2024). Individuals high in SPS tend to process environmental stimuli more deeply and are more easily overwhelmed by sensory input (Greven et al., 2019). Importantly, the interaction between childhood trauma and high SPS may amplify adolescents’ emotional vulnerability, as intense processing of traumatic cues could heighten distress and hinder adaptive regulation (Serafini et al., 2016). This suggests that the coexistence of early trauma and heightened sensitivity may jointly contribute to depressive symptom formation. Adolescents with both high SPS and a history of childhood trauma may engage in excessive internal processing of traumatic events, leading to more intense emotional distress and difficulties in academic or social functioning (Acevedo et al., 2014; Serafini et al., 2017).

Moreover, childhood trauma—particularly trauma inflicted by primary caregivers—can exert long-lasting psychological harm, especially when children lack mature coping mechanisms. Such trauma often leads to the internalization of negative emotions, as children attempt to maintain a positive image of caregivers who are simultaneously the source of harm (Lupien et al., 2009; Sekowski et al., 2020). This internalization may manifest as difficulty identifying and expressing vulnerable emotions such as guilt, shame, or sadness. SPS theory posits that highly sensitive individuals are particularly attuned to the quality of their caregiving environment, rendering them more susceptible to depressive symptoms when exposed to adverse conditions (Jagiellowicz et al., 2016). This is consistent with the “diathesis-stress” model and aligns with Aron and Aron (1997) foundational work on SPS as a trait-based risk factor for emotional disorders.

Conversely, individuals with lower levels of SPS may show reduced emotional and physiological responsiveness to traumatic experiences, thereby exhibiting greater resilience in the face of childhood trauma. SPS is considered a relatively stable temperament trait emerging early in life and shaped by environmental experiences over time. The current findings reinforce the notion that SPS plays a key moderating role in the relationship between childhood trauma and depression and shed light on the differential vulnerability mechanisms involved.

These insights hold practical significance, suggesting that personalized interventions that consider individual differences in sensory sensitivity may be more effective in preventing and treating adolescent depression.

### 4.3 Moderating role of ease of excitation, low sensory threshold, and aesthetic sensitivity

Building upon previous research, this study further investigated the moderating roles of the three distinct dimensions of Sensory Processing Sensitivity (SPS). The dimensional moderation analyses revealed that Ease of Excitation (EOE), Low Sensory Threshold (LST), and Aesthetic Sensitivity (AES) each significantly and positively moderated the relationship between childhood trauma and adolescent depression, thereby supporting Hypothesis 3.

With regard to EOE, which reflects the tendency to become easily overwhelmed by internal or external stimuli, the findings indicate that this dimension is particularly responsive to negative experiences. As both childhood trauma and depression represent adverse psychological conditions, the significant positive moderating effect of EOE may reflect a heightened vulnerability to environmental stressors (Meredith et al., 2016; Vander Elst et al., 2019). Adolescents with high EOE scores may exhibit stronger emotional reactivity to traumatic events, making them more susceptible to the development of depressive symptoms.

In terms of LST, which captures heightened sensitivity to sensory input such as noise, light, or temperature, a similar moderating effect was observed. While LST is not inherently biased toward either positive or negative stimuli, some scholars conceptualize it as a “vulnerability” factor (Assary et al., 2021; Pluess et al., 2018), whereas others interpret it as a “plasticity” trait (Smolewska et al., 2006). In the current study, LST was positively associated with negative outcomes, suggesting that adolescents with high LST scores are more likely to develop depressive symptoms in response to childhood trauma. These findings support the view that LST may represent a context-sensitive trait, amplifying individuals’ reactivity to environmental inputs regardless of valence.

The AES dimension, characterized by heightened aesthetic awareness and deeper cognitive-emotional engagement with stimuli such as music, art, and nature (Smolewska et al., 2006), also showed a significant positive moderating effect in the childhood trauma–depression link. This finding diverges from earlier studies that suggest high AES is associated with emotional warmth and prosocial behavior through the internalization of positive caregiver experiences (Hastings et al., 2015). In contrast, the present results indicate that adolescents with elevated AES levels may also be more vulnerable to negative emotional outcomes in the context of trauma. This divergence may be due to the specific nature of the variables examined in this study—both childhood trauma and depression are inherently negative constructs. Thus, AES may intensify sensitivity to negative emotional stimuli just as it does for positive ones. While previous studies often link AES to adaptive outcomes, such associations may be context-dependent rather than universal.

Moreover, cultural and demographic factors may also account for these discrepancies. Most existing SPS research has been conducted with Western populations, particularly in the UK and the US. By contrast, this study used a Chinese adolescent sample, highlighting the possibility that cultural and ethnic contexts influence how AES manifests and interacts with psychological variables. According to SPS theory, individual sensitivity traits are shaped by evolutionary adaptation as well as by genetic and environmental influences (Aron et al., 2012). Therefore, future research should incorporate cross-cultural perspectives to better understand the multifaceted and context-dependent nature of AES and other SPS dimensions.

In summary, this study provides novel empirical insights into the distinct moderating roles of EOE, LST, and AES in the relationship between childhood trauma and adolescent depression. The findings underscore the importance of examining sensitivity not as a uniform construct but as a multidimensional trait with diverse effects depending on environmental and cultural context. These insights offer promising directions for the development of tailored psychological interventions that take individual sensitivity profiles into account.

### 4.4 Implications

This study contributes significantly to the theoretical understanding of adolescent psychopathology by integrating the concepts of childhood trauma (childhood trauma) and sensory processing sensitivity (SPS) within a moderation framework. The results provide empirical evidence supporting both the diathesis-stress model and the differential susceptibility hypothesis, which posit that individual traits interact with environmental stressors to influence developmental outcomes. By demonstrating that SPS and its dimensions (EOE, LST, AES) moderate the relationship between childhood trauma and depression, the study highlights that vulnerability to early life adversity is not uniform across individuals, but rather shaped by dispositional sensitivity. Although prior studies have linked sensory processing sensitivity with various psychological outcomes, few have specifically explored its moderating effect on the relationship between childhood trauma and depression during adolescence. By addressing this gap, the present study advances current understanding of how individual sensitivity traits interact with early adverse experiences to shape depressive outcomes.

Furthermore, the dimensional analysis of SPS offers a more nuanced view of sensitivity traits, suggesting that different components of SPS may have distinct psychological implications. This dimensional approach encourages future researchers to go beyond global sensitivity scores and investigate the specific roles of EOE, LST, and AES in various psychopathological processes. The study also addresses a notable gap in the existing literature by focusing on a non-Western population, offering cross-cultural insights into how sensitivity traits may operate differently across sociocultural contexts. This cross-cultural extension of SPS theory encourages the development of more inclusive, culturally responsive theoretical models.

From a practical perspective, the findings underscore the importance of individualized mental health assessment and intervention, particularly for adolescents with high sensitivity traits. In school-based and clinical settings, early screening for both trauma exposure and sensitivity characteristics can help identify adolescents who may be at elevated risk for depression. Tailoring intervention programs to these high-risk individuals could significantly improve outcomes. For instance, highly sensitive adolescents may benefit from trauma-informed counseling approaches that incorporate emotion regulation training, mindfulness practices, or environmental modifications to reduce overstimulation.

Educators, school psychologists, and clinicians should also be aware that adolescents with heightened sensitivity—especially in the EOE and LST dimensions—may react more intensely to environmental stressors and require additional emotional support. Meanwhile, those with high AES scores might benefit from creative therapies, such as art or music therapy, which can channel their heightened aesthetic awareness into emotionally expressive and restorative activities.

Future research could further extend these findings by examining other developmental outcomes associated with sensory sensitivity, such as anxiety, emotion regulation, or social withdrawal, which may also mediate or moderate the long-term effects of childhood trauma. Additionally, integrating neurobiological and cross-cultural perspectives could help clarify whether the moderating effects of SPS are universal or shaped by cultural and environmental contexts. Additionally, public health campaigns and prevention strategies could be more effective if they account for variability in sensory processing sensitivity, promoting psychoeducation not only for adolescents but also for parents and teachers, who play critical roles in shaping supportive environments. In sum, this research provides a strong rationale for incorporating sensitivity-informed approaches into adolescent mental health services and education policy.

### 4.5 Limitations

Several limitations should be acknowledged when interpreting the findings and designing future studies. First, the sample was drawn exclusively from two high schools in Yunnan Province, China. This geographically and culturally limited sample may restrict the generalizability of the findings to broader adolescent populations. Future research should incorporate more diverse and representative samples across different regions and cultural backgrounds to enhance external validity.

Second, both childhood trauma and depressive symptoms were assessed using self-report questionnaires, which may be subject to biases such as memory distortion, subjective interpretation, and social desirability effects. The retrospective nature of the trauma measure may further compromise accuracy due to recall bias. To improve validity, future studies should consider using multiple data sources—such as caregiver or teacher reports, school records, or structured clinical interviews—and, where feasible, include objective or clinician-administered assessments of depressive symptoms.

Third, the study employed a cross-sectional design, which limits causal inference and fails to capture the temporal dynamics among childhood trauma, SPS, and depressive symptoms. Longitudinal research is needed to track developmental trajectories and examine how these variables evolve and interact over time.

Addressing these limitations in future research will help to clarify causal mechanisms, enhance measurement precision, and support the development of more robust, context-sensitive models of adolescent mental health.

## 5 Conclusion

This study empirically demonstrated that childhood trauma is significantly associated with adolescent depression. It further revealed that sensory processing sensitivity (SPS)—including its three key dimensions, ease of excitation (EOE), low sensory threshold (LST), and aesthetic sensitivity (AES)—plays a critical moderating role in this relationship. These findings offer important insights into how individual differences in sensitivity traits influence adolescents’ psychological responses to early adverse experiences. By highlighting the moderating effects of SPS, the study contributes to a more nuanced understanding of the interaction between environmental risk factors and dispositional traits in the development of depressive symptoms during adolescence. It enriches theoretical frameworks such as the diathesis-stress model and the differential susceptibility theory, and underscores the importance of considering individual variability when assessing mental health risks. Future research should strive to recruit more diverse and representative samples to enhance the external validity of the findings. Additionally, longitudinal research designs are recommended to clarify the temporal and causal relationships among childhood trauma, sensitivity traits, and depressive outcomes. Such efforts would not only deepen the scientific understanding of developmental psychopathology but also inform the development of personalized, sensitivity-informed mental health interventions tailored to the unique profiles of vulnerable adolescents. Given that childhood trauma remains a central risk factor for numerous psychiatric symptoms, preventive strategies should receive more policy and clinical attention. Schools and community mental health services should implement early screening programs to identify vulnerable children and provide trauma-informed education. Moreover, parental training in emotional communication, safe caregiving, and stress management could play a crucial role in reducing the occurrence and impact of childhood trauma. Developing these preventive frameworks would complement the current study’s emphasis on sensitivity-informed interventions, ensuring more comprehensive support for at-risk youth.

## Data Availability

The raw data supporting the conclusions of this article will be made available by the authors, without undue reservation.
